# EHMTI-0284. Frequency and intensity of headache following vasospasm after SAH

**DOI:** 10.1186/1129-2377-15-S1-C67

**Published:** 2014-09-18

**Authors:** N Zaric, N Milovanovic-Kovacevic, M Savic

**Affiliations:** 1 Neurosonology, Hospital for Cerebrovascular Diseases Sveti Sava, Belgrade, Serbia

## Background

Vasospasm after SAH can begin 3–14 days after the first bleed, most commonly between 7–10days. Symptoms of SAH include a severe headache with a rapid onset, that belongs to symptomatic headaches and is classified in the 6th group IHS classification. The aim of our study was to determine frequency and intensity of the headache following vasospasm after SAH, using TCD for diagnosis of MCA spasm.

## Method

The study included 69 patients (age 55+/-10years) after SAH , admitted to the St Sava Hospital from January 1 to December 31,2012.TCD ultrasound was performed every 24–48 hours to monitor possible development of vasospasm and its gravity. MCA/ICA index and MFVof the M1 branches were measured with TCD and compared with presence of headache, its frequency and intensity established by taking the history from the patients or relatives.

## Results

MFV for M1-MCA were significantly higher in patients with spasm than in those without spasm(p>0.01) and MCA/ICA index was >3. The ROC curve identified the best cut-off point for M1(MFV125cm/s). Those with mild to medium spasm (MFV–M1 < 120cm/s) described headache as a dull lingering pain. Those with severe vasospasm (MFV >200 cm/s ) experienced sudden and strong pain that referred to a "thunderclap headache" which further increased to a headache described as "like being kicked in the head", or the "worst ever" pulsated towards the occiput.

## Conclusion

Our results confirm that frequency rate and intensity of headache is proportionate to vasospasm and that severe headache is most common in patients with vasospasam of high degree, occurring between 3-7 days after SAH.

No conflict of interest.

